# Risks of ovarian, breast, and corpus uteri cancer in women treated with assisted reproductive technology in Great Britain, 1991-2010: data linkage study including 2.2 million person years of observation 

**DOI:** 10.1136/bmj.k2644

**Published:** 2018-07-11

**Authors:** Carrie L Williams, Michael E Jones, Anthony J Swerdlow, Beverley J Botting, Melanie C Davies, Ian Jacobs, Kathryn J Bunch, Michael F G Murphy, Alastair G Sutcliffe

**Affiliations:** 1 UCL Great Ormond Street Institute of Child Health, London, UK; 2 Institute of Cancer Research, London, UK; 3 Institute for Women’s Health, University College London Hospitals, London, UK; 4 University of New South Wales, Sydney, NSW, Australia; 5 National Perinatal Epidemiology Unit, University of Oxford, Oxford, UK; 6 Nuffield Department of Obstetrics and Gynaecology, University of Oxford, Oxford, UK

## Abstract

**Objective:**

To investigate the risks of ovarian, breast, and corpus uteri cancer in women who have had assisted reproduction.

**Design:**

Large, population based, data linkage cohort study.

**Setting and participants:**

All women who had assisted reproduction in Great Britain, 1991-2010, as recorded by the Human Fertilisation and Embryology Authority (HFEA).

**Interventions:**

HFEA fertility records for cohort members were linked to national cancer registrations.

**Main outcome measures:**

Observed first diagnosis of ovarian, breast, and corpus uteri cancer in cohort members were compared with age, sex, and period specific expectation. Standardised incidence ratios (SIRs) were calculated by use of age, sex, and period specific national incidence rates.

**Results:**

255 786 women contributed 2 257 789 person years’ follow-up. No significant increased risk of corpus uteri cancer (164 cancers observed *v* 146.9 cancers expected; SIR 1.12, 95% confidence interval 0.95 to 1.30) was found during an average of 8.8 years’ follow-up. This study found no significantly increased risks of breast cancer overall (2578 *v* 2641.2; SIR 0.98, 0.94 to 1.01) or invasive breast cancer (2272 *v* 2371.4; SIR 0.96, 0.92 to 1.00). An increased risk of in situ breast cancer (291 *v* 253.5; SIR 1.15, 1.02 to 1.29; absolute excess risk (AER) 1.7 cases per 100 000 person years, 95% confidence interval 0.2 to 3.2) was detected, associated with an increasing number of treatment cycles (P=0.03). There was an increased risk of ovarian cancer (405 *v* 291.82; SIR 1.39, 1.26 to 1.53; AER 5.0 cases per 100 000 person years, 3.3 to 6.9), both invasive (264 *v* 188.1; SIR 1.40, 1.24 to 1.58; AER 3.4 cases per 100 000 person years, 2.0 to 4.9) and borderline (141 *v* 103.7; SIR 1.36, 1.15 to 1.60; AER 1.7 cases per 100 000 person years, 0.7 to 2.8). Increased risks of ovarian tumours were limited to women with endometriosis, low parity, or both. This study found no increased risk of any ovarian tumour in women treated because of only male factor or unexplained infertility.

**Conclusions:**

No increased risk of corpus uteri or invasive breast cancer was detected in women who had had assisted reproduction, but increased risks of in situ breast cancer and invasive and borderline ovarian tumours were found in this study. Our results suggest that ovarian tumour risks could be due to patient characteristics, rather than assisted reproduction itself, although both surveillance bias and the effect of treatment are also possibilities. Ongoing monitoring of this population is essential.

## Introduction

Assisted reproduction cycles usually involve exposure to supraphysiological levels of oestradiol, exogenous gonadotropins, and multiple ovarian punctures, all potentially carcinogenic.^1^ ^2^ Most concern surrounds the risks of breast, endometrial, and ovarian cancers after such exposures.^3^ ^4^ ^5^ ^6^ ^7^ ^8^ ^9^ ^10^ ^11^ ^12^ ^13^ ^14^ ^15^ ^16^

Studies investigating breast cancer risks in women who underwent assisted reproduction are inconsistent.^3^ ^4^ ^5^ ^6^ ^7^ ^8^ ^9^ ^10^ ^11^ ^12^ Although some studies have shown an increased risk,^17^ most studies do not show an overall increase of breast cancer in exposed women.^3^ ^4^ ^5^ ^7^ ^8^ ^10^ However, some suggest a possible increased risk within subgroups,^8^ ^9^ including women treated at younger ages^9^ and with multiple cycles.^8^ Most studies investigating endometrial cancer risk in exposed populations have not found a significant increased risk.^3^ ^4^ ^6^ ^7^ ^18^ However, most studies have provided very imprecise estimates due to small sample size and few events.^3^ ^4^ ^6^ ^18^ One study suggested an increased risk of endometrial cancer associated with exposure to gonadotrophins, commonly used as part of assisted reproductive technology.^19^ Some early studies investigating fertility drugs used alone, such as single agent oral clomifene, suggested increased risks of ovarian cancer.^20^ Others found no association between fertility drugs and ovarian cancer risk.^21^ Recent investigations into their use as part of assisted reproduction have generally been more reassuring, but remain inconsistent and at risk of bias.^4^ ^5^ ^11^ Some^13^ ^14^ but not all studies^6^ have found an increase in borderline tumours.

Given previous inconsistent results, small study size, and lack of information on potential confounders, we undertook a population based linkage study in Britain to provide risk estimates for ovarian, breast, and corpus uteri cancer, in a cohort of over 266 000 women undergoing assisted reproduction, with information on potential confounders such as parity and infertility diagnosis.

## Methods

### Study population

We defined assisted reproduction as “treatments or procedures that include in vitro handling of both human oocytes and sperm or embryos, for the purpose of reproduction.”^22^ Records for all women undergoing assisted reproduction from January 1991 to September 2009, and those undergoing the same from October 2009 to December 2010 who gave their prospective consent, in England, Wales, and Scotland were obtained from the Human Fertilisation and Embryology Authority (HFEA).

UK law mandates reporting of all assisted reproduction cycles to the HFEA. For cycles performed before October 2009, research use of these data was permitted, but consent could be withdrawn retrospectively. Fewer than 300 women had done so before this study began (based on the level of reporting detail provided by the HFEA). The study cohort, January 1991 to September 2009, therefore represents about 99.7% of the at-risk population. For cycles performed October 2009 onwards, prospective consent was required. Overall consent was not provided for an estimated 7% of women undergoing assisted reproduction in 1991-2010 (about 20 000 women, based on reports from the HFEA), who were therefore not included in this study, representing a loss of less than 1% of person years’ follow-up (figure S1, supplementary appendix).

### Outcome data

HFEA records were linked to the National Health Service Central Registers of England, Wales, and Scotland (from which emigrations, deaths, and cancer registrations are reported to authorised medical researchers) in a one-off linkage. Completeness and accuracy of these registers have been described.^23^ ^24^ ^25^ Overall, records of 266 787 (95.1%) eligible women were linked (box S1 and figure S1, supplementary appendix). Cancer diagnosis date, topography code (ICD-9/ICD-10 (international classification of diseases, 9th and 10th revisions)), morphology (ICD-O-2/ICD-O-3 (international classification of diseases for oncology, second and third revisions)), and behaviour (ICD-O-2/ICD-O-3) were available where an incident cancer was diagnosed. Women with cancer diagnoses (including non-melanoma skin cancer) recorded before the first treatment year were excluded from analyses. We obtained data relating to potential confounding factors such as infertility diagnosis, parity (as recorded at last treatment cycle completion), and treatment details (including number of stimulated cycles and age at first treatment) for each cohort member from the HFEA database. These data are a combination of patient reported and clinic reported information (table S1). Information regarding infertility diagnoses are reported to the HFEA by assisted reproduction clinics, based on investigations undertaken by that clinic; by the referring clinician; or occasionally by patient self report.

### Statistical analyses

Follow-up was calculated from date of first treatment (estimated as the mid-point of the first treatment year) until the date of any cancer diagnosis, death, emigration, or study end (March 2011), whichever came first. For analyses involving number of cycles, infertility duration, and live and multiple births, person years at risk were calculated from date of last treatment (estimated as mid-point of the last treatment year), because the HFEA did not record intermediate dates required for time dependent analysis. To calculate expected cancers, we multiplied the person years at risk by corresponding national incidence rates (by 5 year age band and individual calendar year) for the general female population of England and Wales. 

Standardised incidence ratios were calculated by the comparison of observed values with expected values. We calculated 95% confidence intervals, two sided P values, and trends assuming a Poisson distribution.^26^ Sensitivity analyses excluded the first 12 months of follow-up, to investigate potential surveillance bias in the period immediately following assisted reproductive treatment (which could arise as a result of treatment and or after-care; supplementary appendix). Absolute excess risks represent an estimate of the increased risk in the study group as compared with the general population and gives a direct measure of excess risk. They are presented per 100 000 person years, with corresponding 95% confidence intervals, based on exact confidence intervals for Poisson counts. Analyses were performed using Stata, version 12.^27^

### Patient involvement and study approval

Representatives from patient support groups were consulted on the original research question, design, and planning of this study. Approval of the study and waiver of the requirement for individual consent were obtained from the UK Health Research Authority Confidentiality Advisory Group and London Research Ethics Committee (references 5.04(b)/10 and 10/H0720/18, respectively). Given the anonymous nature of the final dataset, it is not possible to disseminate results to individual study participants; instead results will be shared with fertility practitioners and clinics through the Human Fertility and Embryology Authority networks.

## Results

### Characteristics of study participants

In total, 255 786 women contributed 2 257 789 person years’ follow-up. Average follow-up was 8.8 years (range 1-19 years), with 105 436 (41%) followed for at least 10 years. Average age at first treatment was 34.5 years. Infertility cause involved at least one female factor in 111 658 women (44%; including endometriosis, ovulatory disorders (predominantly polycystic ovary disease), and tubal disease). Infertility was unexplained in 47 757 (19%) women, and was due only to male factors in 84 871 (33%). Average infertility duration was 4.9 years. Women had 1.8 stimulated cycles on average, with only 20% (n=50 485) having more than two stimulated cycles. About half the study population had at least one live birth after treatment completion (table 1).

**Table 1:** Characteristics of 225 786 women who underwent assisted reproduction in Great Britain, 1991-2010

Characteristic	Total cohort (n=255 786)	Women who developed ovarian, breast, and corpus uteri cancer (n=3155)	Women who did not develop ovarian, breast, and corpus uteri cancer (n=252 631)
Age at first treatment (years; mean (SD))	34.5 (4.8)	36.3 (4.7)	34.5 (4.8)
Age at first treatment (No (%))			
<25 years	5671 (2)	20 (1)	5651 (2)
25-29 years	39 932 (16)	259 (8)	39 673 (16)
30-34 years	92 788 (36)	961 (31)	91 827 (36)
35-39 years	85 868 (34)	1244 (39)	84 624 (34)
40-44 years	28 174 (11)	563 (18)	27 611 (11)
≥45 years	3353 (1)	108 (3)	3245 (1)
Cause of infertility (No (%))			
Any female factor	111 658 (44)	1626 (52)	110 032 (44)
Male factor only	84 871 (33)	915 (29)	83 956 (33)
Unexplained	47 757 (19)	474 (15)	47 283 (19)
Unrecorded	11 500 (5)	140 (4)	11 360 (5)
History of endometriosis (No (%))	18 630 (7)	281 (9)	18 349 (7)
History of tubal disease (No (%))	66 370 (26)	1045 (33)	65 325 (26)
History of ovulatory disorder (No (%))	36 016 (14)	451 (14)	35 565 (14)
Duration of infertility reported at completion of last cycle (years; mean (SD))	4.9 (3.3)	5.6 (3.9)	4.8 (3.3)
No of stimulated cycles (mean (SD))	1.8 (1.2)	1.8 (1.3)	1.8 (1.2)
No of live births at completion of last cycle (mean (SD))	0.6 (0.7)	0.6 (0.7)	0.6 (0.7)
No of live births at completion of last cycle (No (%))			
0	129 217 (51)	1775 (56)	127 442 (50)
1	96 839 (38)	1011 (32)	95 828 (38)
≥2	29 645 (12)	368 (12)	29 277 (11)
Unrecorded	85 (0)	1 (0)	84 (0)
Any multiple birth recorded at completion of last cycle (No (%))	29 366 (11)	304 (10)	29 062 (12)

SD=standard deviation.

### Breast cancer

There was no overall increased risk of breast cancer (2578 observed *v* 2641.2 expected cancers; standardised incidence ratio 0.98 (95% confidence interval 0.94 to 1.01); absolute excess risk −2.8 cases per 100 000 person years (95% confidence interval −7.1 to 1.8); table 2). More than three quarters (76%) of tumours were ductal carcinomas (n=1963), 9% lobular (n=228), 12% other epithelial tumours (n=319), and 3% non-epithelial or unspecified (n=68). There were no significantly raised risks in groups by age at first treatment, infertility duration, number of stimulated cycles, number of live births, and number of multiple births (table 3). 

**Table 2:** Relative and absolute excess risks of cancers of breast, ovary, and corpus uteri among 225 786 women who underwent assisted reproduction in Great Britain, 1991-2010, including and excluding the first year after the start of treatment

Type of cancer	Follow-up (No of person years)	No of observed cancers	No of expected cancers	Standardised incidence ratio (95% CI)	Absolute excess risk (95% CI) per 100 000 person years at risk
**Including first year of follow-up**					
Breast*	2 257 789	2578	2641.2	0.98 (0.94 to 1.01)	−2.8 (−7.1 to 1.8)
Corpus uteri†	2 257 789	164	146.9	1.12 (0.95 to 1.30)	0.8 (−0.3 to 2.0)
Ovary‡	2 257 789	405	291.82	1.39 (1.26 to 1.53)	5.0 (3.3 to 6.9)
**Excluding first year of follow-up**					
Breast*	2 004 121	2384	2501.6	0.95 (0.92 to 0.99)	−5.9 (−10.6 to −1.0)
Corpus uteri†	2 004 121	157	141.79	1.11 (0.94 to 1.30)	0.8 (−0.4 to 2.1)
Ovary‡	2 004 121	356	271.9	1.31 (1.18 to 1.45)	4.2 (2.44 to 6.10)

* Breast cancer=ICD-9 codes 1740-1749, 2330, and 2383; ICD-10 codes C500-C509, D050-D059, and D486.

† Corpus uteri cancer=ICD-9 codes 1820-1828 and ICD-10 code C54.

‡ Ovarian cancer=ICD-9 codes 1830-1839 and 2362; ICD-10 codes C56, C570-C574, C481, C482, and D391.

**Table 3:** Standardised incidence ratios (SIRs) for ovarian, breast, and corpus uteri cancer among 225 786 women who underwent assisted reproduction in Great Britain, 1991-2010, stratified by various factors*

**Factor**	**Follow-up (No of person years)**	Breast cancer†			Corpus uteri cancer‡			Ovarian cancer§	
		No of observed cancers	SIR (95% CI)		No of observed cancers	SIR (95% CI)		No of observed cancers	SIR (95% CI)
**Age at first treatment (years)**									
<25	48 187	14	1.32 (0.72 to 2.21)		0	0.00 (0.00 to 6.97)		6	2.21 (0.81 to 4.80)
25-29	381 964	185	0.92 (0.79 to 1.06)		10	1.24 (0.60 to 2.29)		64	2.16 (1.67 to 2.76)
30-34	866 351	774	0.95 (0.89 to 1.02)		43	1.19 (0.86 to 1.60)		142	1.52 (1.28 to 1.80)
35-39	714 056	1033	0.97 (0.91 to 1.03)		72	1.22 (0.96 to 1.54)		134	1.23 (1.03 to 1.45)
40-44	218 767	479	1.02 (0.93 to 1.12)		33	0.96 (0.66 to 1.35)		50	1.05 (0.78 to 1.38)
≥45	28 463	93	1.09 (0.89 to 1.34)		6	0.68 (0.25 to 1.48)		9	0.97 (0.45 to 1.85)
Trend across categories	—	P=0.13			P=0.28			P<0.001	
**Infertility cause**									
Any female factor	1 109 593	1279	0.95 (0.90 to 1.00)		97	1.25 (1.02 to 1.53)		246	1.66 (1.46 to 1.88)
Male factor only	757 063	774	0.92 (0.86 to 0.99)		41	0.91 (0.65 to 1.24)		98	1.05 (0.85 to 1.27)
Unexplained	326 495	416	1.10 (1.00 to 1.21)		16	0.78 (0.45 to 1.27)		40	0.96 (0.69 to 1.31)
Unrecorded	64 638	109	1.49 (1.24 to 1.80)		10	2.53 (1.21 to 4.66)		21	2.59 (1.60 to 3.95)
**History of endometriosis **									
Yes	181 279	214	0.98 (0.86 to 1.12)		9	0.75 (0.35 to 1.43)		55	2.31 (1.74 to 3.01)
No	2 076 509	2364	0.98 (0.94 to 1.02)		155	1.15 (0.98 to 1.34)		350	1.31 (1.17 to 1.45)
**History of tubal disease **									
Yes	710 522	826	0.96 (0.90 to 1.03)		59	1.23 (0.93 to 1.58)		158	1.68 (1.43 to 1.97)
No	1 547 266	1752	0.98 (0.94 to 1.03)		105	1.06 (0.87 to 1.29)		247	1.25 (1.10 to 1.41)
**History of ovulatory problems **									
Yes	311 523	357	0.92 (0.83 to 1.02)		39	1.59 (1.13 to 2.17)		55	1.28 (0.97 to 1.67)
No	1 946 265	2221	0.99 (0.95 to 1.03)		125	1.02 (0.85 to 1.21)		350	1.41 (1.26 to 1.56)
**Duration of infertility at last cycle (years) **									
<2	133 067	171	0.95 (0.82 to 1.11)		6	0.55 (0.20 to 1.20)		28	1.44 (0.96 to 2.09)
2-3	439 560	527	1.05 (0.96 to 1.14)		23	0.82 (0.52 to 1.23)		73	1.30 (1.02 to 1.64)
4-5	447 739	520	0.99 (0.90 to 1.07)		30	1.03 (0.70 to 1.47)		74	1.27 (1.00 to 1.60)
6-7	271 583	316	0.91 (0.82 to 1.02)		27	1.38 (0.91 to 2.01)		60	1.61 (1.23 to 2.07)
8-9	151 580	197	0.95 (0.83 to 1.10)		16	1.34 (0.77 to 2.18)		36	1.64 (1.15 to 2.27)
≥10	209 751	322	0.95 (0.85 to 1.05)		37	1.68 (1.18 to 2.31)		57	1.60 (1.21 to 2.08)
Unrecorded	324 953	404	1.07 (0.97 to 1.18)		18	0.92 (0.54 to 1.45)		42	1.02 (0.74 to 1.38)
Trend across categories	—	P=0.20			P<0.001			P=0.15	
**Total No of stimulated cycles**									
0 (“natural cycle” only)	90 973	142	0.88 (0.74 to 1.04)		8	0.66 (0.28 to 1.29)		17	0.99 (0.58 to 1.59)
1	1 041 791	1203	0.98 (0.92 to 1.03)		89	1.29 (1.04 to 1.59)		196	1.44 (1.25 to 1.66)
2	473 125	585	1.01 (0.93 to 1.09)		29	0.91 (0.61 to 1.30)		87	1.38 (1.10 to 1.70)
3-4	306 137	420	1.03 (0.93 to 1.13)		24	1.06 (0.68 to 1.58)		53	1.23 (0.92 to 1.60)
≥5	66 149	107	1.08 (0.89 to 1.31)		7	1.24 (0.50 to 2.55)		17	1.67 (0.97 to 2.67)
Trend across categories	—	P=0.07			P=0.93			P=0.86	
**Total number of live births at last cycle completion **									
0	1 009 134	1299	0.99 (0.93 to 1.04)		122	1.61 (1.34 to 1.92)		222	1.57 (1.37 to 1.79)
1	718 998	843	1.03 (0.96 to 1.10)		24	0.53 (0.34 to 0.79)		114	1.25 (1.03 to 1.50)
≥2	249 685	314	0.92 (0.82 to 1.03)		11	0.54 (0.27 to 0.96)		34	0.93 (0.64 to 1.30)
Unrecorded	414	1	1.82 (0.05 to 10.13)		0	0.00 (0.00 to 99.86)		0	0.00 (0.00 to 49.93)
Trend across categories	—	P=0.56			P<0.001			P=0.001	
**Any multiple birth as recorded at last cycle completion **									
Yes	232 824	258	1.10 (0.97 to 1.24)		5	0.42 (0.14 to 0.99)		33	1.23 (0.85 to 1.73)
No	1 745 409	2199	0.98 (0.94 to 1.02)		152	1.17 (1.00 to 1.38)		337	1.39 (1.24 to 1.54)
**Time since last treatment (years) **									
0-3	687 180	525	1.04 (0.95 to 1.13)		28	1.39 (0.92 to 2.00)		99	1.54 (1.25 to 1.88)
3-6	486 191	529	1.04 (0.95 to 1.13)		29	1.28 (0.85 to 1.83)		73	1.27 (1.00 to 1.60)
6-10	444 324	657	1.00 (0.93 to 1.08)		38	1.07 (0.76 to 1.47)		84	1.24 (0.99 to 1.53)
10-15	296 445	590	0.93 (0.86 to 1.01)		45	0.99 (0.72 to 1.33)		86	1.39 (1.11 to 1.71)
≥15	64 091	156	0.86 (0.73 to 1.01)		17	0.98 (0.57 to 1.57)		28	1.57 (1.05 to 2.27)
Trend across categories	—	P=0.01			P=0.12			P=0.74	

* See supplementary appendix for results excluding the first 12 months of follow-up.

† Breast cancer=ICD-9 codes 1740-1749, 2330, and 2383; ICD-10 codes C500-C509, D050-D059, and D486.

‡ Corpus uteri cancer=ICD-9 codes 1820-1828 and ICD-10 code C54.

§ Ovarian cancer=ICD-9 codes 1830-1839 and 2362; ICD-10 codes C56, C570-C574, C481, C482, and D391.

We found significant risk reductions with increasing duration since treatment completion (P=0.01; table 3), and in women with any female factor or only male factor infertility (table 3), but no difference between risks at premenopausal and postmenopausal ages separately (age <50 years, standardised incidence ratio 0.98 (95% confidence interval 0.94 to 1.02); ≥50 years, 0.97 (0.89 to 1.06); data not shown). After exclusion of the first 12 months of follow-up, breast cancer risk was significantly reduced compared with age standardised expectation (standardised incidence ratio 0.95 (0.92 to 0.99), P=0.02; supplementary appendix ). There was no increased risk of invasive breast cancer (standardised incidence ratio 0.96 (0.92 to 1.00); absolute excess risk −4.4 cases per 100 000 person years (95% confidence interval −8.5 to −0.2); table 4), but a small increased risk of in situ breast cancer (291 cancers observed *v* 253.5 cancers expected, standardised incidence ratio 1.15 (1.02 to 1.29); absolute excess risk 1.7 cases per 100 000 person years (0.2 to 3.2); table 4), which was associated with the number of treatment cycles (P=0.03). Exclusion of the first 12 months of follow-up did not substantially change results for in situ breast cancer risk (table S5, supplementary appendix).

**Table 4:** Standardised incidence ratios (SIRs) for invasive and in situ breast cancer and invasive and borderline tumours of the ovary among 255 786 women who underwent assisted reproduction in Great Britain, 1991-2010, stratified by various factors*

Factor	Follow-up (No of person years)	Invasive breast cancer†			In situ breast cancer‡			Invasive ovarian tumours§			Borderline ovarian tumours¶	
		No of observed cancers	SIR (95% CI)		No of observed cancers	SIR (95% CI)		No of observed cancers	SIR (95% CI)		No of observed cancers	SIR (95% CI)
Overall	2 257 789	2272	0.96 (0.92 to 1.00)		291	1.15 (1.02 to 1.29)		264	1.40 (1.24 to 1.58)		141	1.36 (1.15 to 1.60)
Age at first treatment (years)												
<25	48 187	14	1.43 (0.78 to 2.39)		0	0.00 (0.00 to 4.34)		<5	**		<5	**
25-29	38 964	168	0.91 (0.78 to 1.06)		16	1.10 (0.63 to 1.78)		35	2.33 (1.63 to 3.25)		29	1.98 (1.33 to 2.85)
30-34	866 351	685	0.92 (0.86 to 1.00)		85	1.27 (1.02 to 1.57)		81	1.46 (1.16 to 1.82)		61	1.61 (1.23 to 2.07)
35-39	714 056	925	0.97 (0.91 to 1.04)		100	0.94 (0.77 to 1.15)		97	1.32 (1.07 to 1.61)		37	1.04 (0.73 to 1.43)
40-44	218 767	411	1.00 0.90 to 1.10)		66	1.23 (0.95 to 1.56)		40	1.13 (0.80 to 1.53)		10	0.82 (0.39 to 1.50)
≥45	28 463	69	0.94 (0.73 to 1.19)		24	2.12 (1.36 to 3.15)		<10	**		<5	**
Trend across categories	—	P=0.30			P=0.47			P=0.02			P<0.001	
Infertility cause												
Any female factor	1 109 593	1118	0.92 (0.87 to 0.98)		151	1.14 (0.97 to 1.34)		161	1.66 (1.41 to 1.94)		85	1.66 (1.33 to 2.05)
Male factor only	757 063	676	0.89 (0.83 to 0.96)		93	1.18 (0.95 to 1.44)		65	1.09 (0.84 to 1.39)		33	0.96 (0.66 to 1.35)
Unexplained	326 495	374	1.10 (0.99 to 1.22)		42	1.18 (0.85 to 1.59)		26	0.98 (0.64 to 1.44)		14	0.92 (0.50 to 1.55)
Unrecorded	64 638	104	1.58 (1.30 to 1.92)		5	0.73 (0.24 to 1.70)		12	2.35 (1.21 to 4.10)		9	3.00 (1.37 to 5.70)
History of endometriosis												
Yes	181 279	186	0.95 (0.82 to 1.10)		26	1.25 (0.81 to 1.83)		38	2.47 (1.75 to 3.39)		17	2.03 (1.18 to 3.25)
No	2 076 509	2086	0.96 (0.92 to 1.00)		265	1.14 (1.01 to 1.28)		226	1.31 (1.14 to 1.49)		124	1.30 (1.08 to 1.55)
History of tubal disease												
Yes	710 522	725	0.94 (0.87 to 1.01)		92	1.11 (0.89 to 1.36)		105	1.71 (1.40 to 2.08)		53	1.62 (1.21 to 2.12)
No	1 547 266	1547	0.97 (0.92 to 1.01)		199	1.17 (1.01 to 1.34)		159	1.25 (1.07 to 1.46)		88	1.24 (0.99 to 1.53)
History of ovulatory problems												
Yes	311 523	315	0.91 (0.81 to 1.02)		41	1.05 (0.75 to 1.42)		33	1.16 (0.80 to 1.63)		22	1.52 (0.96 to 2.31)
No	1 946 265	1957	0.97 (0.92 to 1.01)		250	1.17 (1.03 to 1.32)		231	1.45 (1.27 to 1.65)		119	1.33 (1.11 to 1.60)
Duration of infertility at last cycle (years)												
<2	133 067	156	0.97 (0.83 to 1.14)		15	0.82 (0.46 to 1.35)		16	1.23 (0.70 to 1.99)		12	1.89 (0.98 to 3.30)
2-3	439 560	464	1.03 (0.94 to 1.13)		61	1.26 (0.97 to 1.62)		53	1.48 (1.11 to 1.93)		20	0.99 (0.61 to 1.53)
4-5	447 739	461	0.97 (0.89 to 1.07)		52	1.03 (0.77 to 1.35)		53	1.42 (1.06 to 1.85)		21	1.02 (0.63 to 1.55)
6-7	271 583	278	0.90 (0.79 to 1.01)		35	1.03 (0.72 to 1.44)		40	1.63 (1.16 to 2.21)		20	1.57 (0.96 to 2.42)
8-9	151 580	169	0.92 (0.78 to 1.06)		27	1.31 (0.86 to 1.91)		27	1.84 (1.21 to 2.67)		9	1.24 (0.57 to 2.36)
≥10	209 751	279	0.92 (0.82 to 1.04)		42	1.15 (0.83 to 1.56)		40	1.60 (1.14 to 2.18)		17	1.61 (0.94 to 2.58)
Unrecorded	324 953	355	1.05 (0.94 to 1.16)		48	1.37 (1.01 to 1.82)		25	0.97 (0.63 to 1.43)		17	1.12 (0.65 to 1.79)
Trend across categories	—	P=0.11			P=0.58			P=0.25			P=0.42	
Total number of stimulated cycles												
0 (“natural cycle” only)	90 973	121	0.85 (0.71 to 1.02)		21	1.14 (0.71 to 1.74)		13	1.04 (0.55 to 1.78)		<5	**
1	1 041 791	1073	0.97 (0.91 to 1.03)		121	1.02 (0.85 to 1.22)		129	1.47 (1.23 to 1.75)		67	1.39 (1.08 to 1.77)
2	473 125	512	0.98 (0.90 to 1.07)		70	1.25 (0.97 to 1.58)		56	1.37 (1.03 to 1.78)		31	1.40 (0.95 to 1.98)
3-4	306 137	371	1.01 (0.92 to 1.12)		47	1.18 (0.87 to 1.57)		42	1.48 (1.06 to 1.99)		11	0.75 (0.37 to 1.33)
≥5	66 149	85	0.96 (0.77 to 1.91)		21	2.11 (1.31 to 3.23)		14	2.04 (1.11 to 3.42)		<5	**
Trend across categories	—	P=0.27			P=0.03			P=0.29			P=0.18	
Total number of live births after last treatment												
0	1 009 134	1154	0.98 (0.92 to 1.04)		135	1.04 (0.87 to 1.23)		156	1.67 (1.42 to 1.95)		66	1.38 (1.07 to 1.75)
1	718 998	732	0.99 (0.92 to 1.07)		107	1.37 (1.12 to 1.65)		78	1.34 (1.06 to 1.67)		36	1.09 (0.76 to 1.51)
≥2	249 685	276	0.90 (0.80 to 1.02)		37	1.07 (0.76 to 1.48)		20	0.81 (0.50 to 1.26)		14	1.16 (0.63 to 1.95)
Unrecorded	414	0	0.00		1	20.00 (0.51 to 111.43)		0	0.00 (0.00 to 74.89)		0	0.0 (0.0 to 149.79)
Trend across categories	—	P=0.37			P=0.32			P=0.001			P=0.34	
Any multiple birth recorded												
Yes	232 824	234	1.10 (0.97 to 1.25)		22	1.05 (0.66 to 1.58)		22	1.34 (0.84 to 2.03)		11	1.06 (0.53 to 1.90)
No	1 745 409	1928	0.96 (0.92 to 1.00)		258	1.16 (1.02 to 1.31)		232	1.45 (1.27 to 1.65)		105	1.27 (1.04 to 1.54)
Time since last treatment (years)												
0-3	687 180	488	1.05 (0.96 to 1.15)		37	1.06 (0.71 to 1.39)		62	1.73 (1.33 to 2.22)		37	1.30 (0.92 to 1.79)
3-6	486 191	476	1.03 (0.94 to 1.12)		51	1.24 (0.93 to 1.63)		45	1.27 (0.93 to 1.71)		28	1.27 (0.85 to 1.84)
6-10	444 324	556	0.94 (0.87 to 1.02)		95	1.52 (1.23 to 1.85)		63	1.37 (1.05 to 1.75)		21	0.96 (0.59 to 1.46)
≥10	296 445	510	0.93 (0.85 to 1.01)		75	0.98 (0.77 to 1.22)		63	1.38 (1.06 to 1.77)		23	1.39 (0.88 to 2.08)
≥15	64 091	132	0.86 (0.72 to 1.02)		22	0.85 (0.54 to 1.29)		21	1.52 (0.94 to 2.32)		7	1.75 (0.70 to 3.60)
Trend across categories	—	P=0.005			P=0.29			P=0.44			P=0.84	

* See supplementary appendix for results excluding the first 12 months of follow-up.

† Invasive breast cancer=ICD-9 codes 1740-1749 and ICD-10 codes C500-C509.

‡ In situ breast cancer=ICD-9 code 2330 and ICD-10 code D050-D059.

§ Invasive ovarian tumours=ICD-9 codes 1830-1839 (excluding morphology codes 8442/8451/8462/8472/8473) and 2362; ICD-10 codes C56, C570-C574, C481, and C482 (excluding morphology codes 8442/8451/8462/8472/8473).

¶ Borderline ovarian tumours=ICD-9 code 1830 (with morphology codes 8442/8451/8462/8472/8473) and ICD-10 codes D391 and C56 (with morphology codes 8442/8451/8462/8472/8473).

** Data suppressed to comply with data disclosure regulations where cells relate to small numbers of individuals. None of the standardised incidence ratios for affected cells approached significance.

### Carcinoma of the corpus uteri

Risk of corpus uteri cancer was not significantly raised (standardised incidence ratio 1.12 (95% confidence interval 0.95 to 1.30); absolute excess risk 0.8 cases per 100 000 person years (95% confidence interval −0.3 to 2.0); table 2). Over 92% (n=152) of corpus uteri tumours were epithelial, 70% (n=107) of which were endometrioid; 8% were non-epithelial or unspecified (n=12). We found a significantly increased risk of corpus uteri cancer in women with an ovulatory disorder (standardised incidence ratio 1.59 (1.13 to 2.17); table 3). There was a highly significant trend of increasing risk with decreased parity (P<0.001), and a significantly decreased risk with women having a multiple birth (standardised incidence ratio 0.42 (0.14 to 0.99); table 3). No significant variation in risk was noted with number of cycles (P=0.93), age at first treatment (P=0.28) or duration since treatment completion (P=0.12). Exclusion of the first 12 months of follow-up did not substantially change results (table S3, supplementary appendix).

### Ovarian cancer

An overall increased risk of ovarian cancer was observed in our study population (standardised incidence ratio 1.39 (95% confidence interval 1.26 to 1.53); absolute excess risk 5.0 cases per 100 000 person years (95% confidence interval 3.3 to 6.9); table 2). Increased risks were seen across most age groups at first treatment, but there was a highly significant trend of increasing risk with decreasing age at first treatment (P<0.001; table 3). Significantly increased risks were found in women who had any diagnosis of female factor infertility (standardised incidence ratio 1.66 (1.46 to 1.88)), particularly endometriosis (2.31 (1.74 to 3.01)) or tubal disease (1.68 (1.43 to 1.97); table 3). No increased risk was seen where infertility was male factor only (standardised incidence ratio 1.05 (0.85 to 1.27)) or unexplained (0.96 (0.69 to 1.31); table 3). There was a significant trend of decreasing risk with increasing number of live births (P=0.001), and women remaining nulliparous after treatment completion conferred the highest risk (standardised incidence ratio 1.57 (1.37 to 1.79); table 3). No increased risk was seen with increasing infertility duration (P=0.15), number of cycles (P=0.86), or duration since treatment completion (P=0.74). Exclusion of the first 12 months of follow-up did not substantially change results (table S3, supplementary appendix).

When tumours were classified as invasive or borderline, significant excesses of both were noted (264 observed *v* 188.1expected cancers, standardised incidence ratio 1.40 (95% confidence interval 1.24 to 1.58), absolute excess risk 3.4 cases per 100 000 person years (95% confidence interval 2.0 to 4.9) and 141 *v* 103.7, 1.36 (1.15 to 1.60), 1.7 cases per 100 000 person years (0.7 to 2.8), respectively; table 4).

### Invasive ovarian tumours

There was a significant trend of increasing risk of invasive ovarian tumours with decreasing age at first treatment (P=0.02; table 4). Significantly increased risks were detected in women who had any diagnosis of female factor infertility (standardised incidence ratio 1.66 (95% confidence interval 1.41 to 1.94)), particularly endometriosis (2.47 (1.75 to 3.39)) or tubal disease (1.71 (1.40 to 2.08); table 4). Risk significantly decreased with increasing parity (P=0.001), and women nulliparous after treatment completion were at greatest risk (1.67 (1.42 to 1.95); table 4). We saw no significant variation in risk with number of cycles (P=0.29), infertility duration (P=0.25), or duration since treatment completion (P=0.44), nor was risk raised in women treated for male factor only infertility (1.09 (0.84 to 1.39); table 4). A third of invasive ovarian tumours were serous (n=87), 25% endometrioid (n=66), 8% mucinous (n=22), 17% other or unspecified epithelial tumours (n=45), and 17% non-epithelial or unspecified (n=44). Exclusion of the first 12 months of follow-up did not substantially change results (table S4, supplementary appendix).

### Borderline ovarian tumours

Significantly increased risks of borderline ovarian tumour was associated with decreasing age at first treatment (P<0.001) and any diagnosis of female factor infertility (standardised incidence ratio 1.66 (95% confidence interval 1.33 to 2.05)), particularly endometriosis (2.03 (1.18 to 3.25)) or tubal disease (1.62 (1.21 to 2.12); table 4). Risk did not change significantly with number of cycles (P=0.18), parity (P=0.34), infertility duration (P=0.42), or duration since treatment completion (P=0.84), nor was risk raised in women treated for male factor only infertility (0.96 (0.66 to 1.35); table 4). Close to half of borderline tumours were serous (n=64), 34% mucinous (n=48), less than 2% endometrioid (n<5), less than 2% other or unspecified epithelial tumours (n<5), and 18% non-epithelial or unspecified (n=25). Exclusion of the first 12 months of follow-up reduced the risk of borderline ovarian tumours (1.19 (0.98 to 1.43); table S4, supplementary appendix) and risk in relation to endometriosis (1.57 (0.81 to 2.73); table S4, supplementary appendix).

### Ovarian cancer risk stratified by risk factors

Parous women who did not have a diagnosis of endometriosis did not have an increased risk of ovarian cancer overall (standardised incidence ratio 1.03 (95% confidence interval 0.86 to 1.22)), invasive tumours (1.03 (0.82 to 1.27)), or borderline tumours (1.02 (0.75 to 1.35); table 5). Risks of all types of ovarian cancer were raised in nulliparous women who did not have a diagnosis of endometriosis but to a lesser extent than in parous women with endometriosis (table 5). Women who were nulliparous with a diagnosis of endometriosis had greater risk of invasive ovarian tumour (2.64 (1.69 to 3.93); table 5) than women with just one of these risk factors. By contrast, nulliparous women with endometriosis had no significant risk of a borderline tumour (1.47 (0.59 to 3.04)), although nulliparity and endometriosis were each separately associated with increased risk (table 5). The significant association between decreasing age at first treatment and increasing risk of invasive ovarian tumour was present in women with at least one of endometriosis or nulliparity (P<0.001), but not in those without either (P=0.62); however, these analyses were based on small numbers (table S6, supplementary appendix).

**Table 5:** Standardised incidence ratios (SIRs) for all, invasive, and borderline ovarian cancers among 225 786 women who underwent assisted reproduction in Great Britain, 1991-2010, by presence or absence of known risk factors endometriosis and nulliparity

Factor	Follow-up (No of person years)	Type of ovarian cancer							
		All ovarian cancer*			Invasive cancer†			Borderline tumours‡	
		No of observed cancers	SIR (95% CI)		No of observed cancers	SIR (95% CI)		No of observed cancers	SIR (95% CI)
No diagnosis of endometriosis and at least one birth recorded by treatment completion	1 036 996	133	1.03 (0.86 to 1.22)		85	1.03 (0.82 to 1.27)		48	1.02 (0.75 to 1.35)
No diagnosis of endometriosis and no births recorded by treatment completion	1 039 514	217	1.57 (1.37 to 1.79)		141	1.56 (1.32 to 1.84)		76	1.57 (1.24 to 1.97)
Diagnosis of endometriosis and at least one birth recorded by treatment completion	79 870	24	2.41 (1.55 to 3.59)		14	2.22 (1.21 to 3.72)		10	2.76 (1.33 to 5.08)
Diagnosis of endometriosis and no birth recorded by treatment completion	101 368	31	2.24 (1.52 to 3.18)		24	2.64 (1.69 to 3.93)		7	1.47 (0.59 to 3.04)

* Ovarian cancer=ICD-9 codes 1830-1839 and 2362; ICD-10 codes C56, C570-C574, C481, C482, and D391.

† Invasive ovarian tumours=ICD-9 codes 1830-1839 (excluding morphology codes 8442/8451/8462/8472/8473) and 2362; ICD-10 codes C56, C570-C574, C481, and C482 (excluding morphology codes 8442/8451/8462/8472/8473).

‡ Borderline ovarian tumours=ICD-9 code 1830 (with morphology codes 8442/8451/8462/8472/8473) and ICD-10 codes D391 and C56 (with morphology codes 8442/8451/8462/8472/8473).

## Discussion

Assisted reproduction is practiced worldwide, and more than five million children have been born as a result.^28^ It is important to establish related disease risks for affected individuals, public health systems, and for counselling of potential patients. In this large population based cohort, we found no overall increased risk of breast cancer associated with assisted reproduction, consistent with most^3^ ^4^ ^5^ ^6^ ^7^ ^9^ ^10^ but not all^12^ published studies. We found no significant association between the risk of breast cancer and age at first treatment, in contrast to a small number of earlier studies.^8^ ^9^ ^29^ Reasons for significant decreases in breast cancer risk seen in some subanalyses—such as women who had assisted reproduction for female factor infertility—are unclear, but could reflect beneficial levels of lifestyle related risk factors for breast cancer.^30^ ^31^ However, details of these risk factors and also age at first birth were not available. 

Menopausal status did not seem to account for the significant reduction in risk with increasing follow-up. Despite no increased risk of invasive breast tumours, there was a significant increase in in situ tumours which was significantly associated with increasing number of stimulated cycles. Interpretation of these findings is challenging: the significant association with increasing number of cycles suggests a causal association, yet there was no overall increased risk of breast cancer. Other potential explanations include surveillance bias, chance, and potential confounding by factors such as socioeconomic status, given that most cycles within our cohort were privately funded. To our knowledge, this study is the first to analyse risks of in situ and invasive breast cancers after assisted reproduction separately, so there are no previous data with which to compare.

Risk of corpus uteri cancer overall was not raised in our study. Women with the known risk factor of nulliparity^32^ and those with a history of ovulatory problems (mainly the known risk factor polycystic ovary disease^33^) were found to have an increased risk of corpus uteri cancer. Most similar studies contained few events.^3^ ^5^ ^6^ The largest studies included 15^4^ and 49 cases^7^ of endometrial cancer in women after assisted reproduction, and neither suggested an increased risk.

We found an excess of ovarian cancer compared with age standardised expectation. Significant increases were observed for both invasive and borderline tumours, but were not seen in women without the known risk factors of endometriosis^34^ ^35^ and nulliparity.^35^ Ovarian cancer risks were not associated with number of treatment cycles, time since treatment completion, or male factor or unexplained infertility, which argues against a causal role for assisted reproduction procedures. However, we did find a significant association between age at first treatment and risk of all, invasive, and borderline ovarian cancers. Previous studies investigating invasive ovarian tumour risk after assisted reproduction^3^ ^4^ ^5^ ^6^ ^7^ ^11^ ^13^ ^15^ ^16^ have generally found increased risks in comparison with the general population when potential confounding effects of infertility have not been considered,^16^ but not when such factors were taken into account.^3^ ^4^ ^11^ ^16^ While our study compared cancer incidence with that in the general population (standardised for age and calendar year), it had sufficient size to stratify by potential confounding factors and thereby to investigate characteristics of associations. We found an increased risk of borderline ovarian tumour in women having assisted reproduction compared with the general population. As with invasive ovarian tumours, this increased risk was not seen in parous women without endometriosis. Few studies have investigated the risk of borderline ovarian cancer in women after assisted reproduction,^6^ ^13^ ^14^ but increased risks have been found in studies in the Netherlands^13^ and Australia.^14^

Although the increased risk in borderline ovarian cancer in women with assisted reproduction could be genuine, it could also be due to surveillance bias. The frequency of borderline tumour diagnosis is increased in ovarian cancer screening studies using ultrasound,^36^ and women who have undergone assisted reproduction might have more frequent ultrasound scans after treatment than the general population. This potential bias is supported by the reduction in overall risk after we excluded the first 12 months of follow-up. However, sensitivity analyses looking at time to diagnosis, age at diagnosis, diagnosis in women of high socioeconomic status, and clinical presentation in other studies suggested surveillance bias an unlikely cause of increased risks.^13^ ^14^ We are not able to further differentiate surveillance bias from a genuine increase in borderline tumours. Women with unrecorded cause of infertility had significantly increased rates of breast, ovarian, and corpus uteri cancers. Reasons are unclear but might include reverse causality (box S2, supplementary appendix).

### Strengths and limitations of the study

Most studies investigating risks of cancer in women after assisted reproduction have been small,^6^ ^8^ with few events and short follow-up.^4^ ^5^ ^6^ ^7^ Two of the largest studies published so far include 67 608^4^ and 113 226^7^ women treated with assisted reproduction. Systematic reviews have included at most 70 753 treated women for analyses of breast cancer risk,^10^ 79 143 for ovarian cancer risk,^16^ and 118 320 for analysis of all gynaecological cancer risk.^37^ Our study comprised over 250 000 treated women, including almost 65 000 person years of follow-up for at least 15 years beyond last treatment with an average follow-up of 8.8 years and a maximum follow-up of 19 years (table S2, supplementary appendix). However, we cannot exclude the possibility of different risk profiles for any studied cancer on longer follow-up, at ages when most reproductive related cancers occur.^35^

Women treated with assisted reproduction are likely to differ from the general population in their parity, age at first birth, age at menopause, and the incidence of predisposing conditions such as endometriosis. More information on these and other factors (eg, socioeconomic status, oral contraceptive use, body mass index, and breastfeeding) would be useful. Comparison to women with untreated infertility problems might have been beneficial, although interpretational problems would remain because of potential selection factors for treatment. Although our study was not able to compare with such a group as some smaller studies have done,^4^ ^13^ ^14^ large study size enabled us to stratify for some important potential confounders and draw inferences despite using general population rates as our comparator. While comparator rates do include cohort participants, less than 5% of the population of reproductive age women underwent assisted reproduction, and our standardised incidence ratios were generally lower than 2.0; therefore, resulting bias will have been minimal.^38^

Infertility diagnoses were reported by treating fertility clinics to the HFEA. No data were available about how such diagnoses were made. Further details of specific treatments could have enabled detailed analysis of risk by treatment type. However, over our 19 year study period, ovarian stimulation regimens as part of assisted reproductive cycles have been relatively constant, with the majority of advances leading to better success rates having occurred in assisted reproduction laboratories. Gonadotrophin injections have been used for ovarian stimulation and human chorionic gonadotropin for triggering ovulation throughout the study period, and while new highly purified and recombinant versions have been used in more recent years, they are essentially equivalent. Clomifene citrate was used as additional ovarian stimulation in the pioneering years of assisted reproduction treatment, but this was uncommon by 1991. Downregulated cycles using GnRH (gonadotrophin releasing hormone) agonists were standard by 1991 and not replaced by GnRH antagonists as standard until after the study period. Progesterone support was used throughout the study period. The number of ovarian punctures per cycle and information about fertility treatment before assisted reproduction were not available.

### Conclusions and implications

In this large, national population based study of British women after assisted reproductive technology treatment, no increased risk of corpus uteri or invasive breast cancer was detected. There was an increased risk of in situ breast cancer associated with increasing number of treatment cycles. We also observed an excess of all types of ovarian cancer. However, our results suggest that this finding is more likely due to underlying patient characteristics, rather than assisted reproduction itself. We were not able to distinguish between a genuine increase in risk of borderline ovarian tumours and other explanations including surveillance bias. Further investigation of this and longer follow-up is warranted to continue monitoring these important outcomes in this ever growing population.

**What is already known on this topic**

Risks of reproductive cancers in women who have undergone assisted reproduction procedures are uncertain
Some previous studies have suggested a possible increased risk of breast cancer in women treated at younger ages and with multiple cycles; previous studies investigating endometrial cancer risk are underpowered
Early studies suggested increased risks of ovarian cancer in these women, while more recent studies are more reassuring, although inconsistent, regarding any increase in borderline ovarian tumours

**What this study adds**

In this large population based study, endometrial cancer was not increased in women who had assisted reproduction in Great Britain in 1991-2010 when compared with the general population
The risk of breast cancer overall and of invasive breast cancer was not increased, but there was a small increased risk of in situ breast cancer
Increased risks of ovarian cancer, both invasive and borderline, were observed but limited to women with other known risk factors; these findings require further investigation

## Web extra

Extra material supplied by authors

**Supplementary appendix:** figures S1 and S2, boxes S1 and S2, and tables S1-S6

## Notes

Contributors: CLW jointly conceptualised and designed the study, devised the linkage protocol, supervised the linkage, carried out the analysis, interpreted data, drafted the initial manuscript, and approved the final manuscript as submitted. MEJ jointly conceptualised and designed the study, jointly supervised the analysis, interpreted data, reviewed and revised the manuscript, and approved the final manuscript as submitted. AJS jointly conceptualised and designed the study, interpreted data, reviewed and revised the manuscript, and approved the final manuscript as submitted. BJB jointly conceptualised and designed the study, jointly supervised the analysis, interpreted data, reviewed and revised the manuscript, and approved the final manuscript as submitted. MCD jointly conceptualised and designed the study, interpreted data, reviewed and revised the manuscript, and approved the final manuscript as submitted. IJ jointly conceptualised and designed the study, interpreted data, reviewed and revised the manuscript, and approved the final manuscript as submitted. KJB jointly conceptualised and designed the study, interpreted data, reviewed and revised the manuscript, and approved the final manuscript as submitted. MFGM jointly conceptualised and designed the study, interpreted data, reviewed and revised the manuscript, and approved the final manuscript as submitted. AGS jointly conceptualised and designed the study, interpreted data, reviewed and revised the manuscript, and had the final decision over submission of the approved the final manuscript. All authors had access to the data and take responsibility for the integrity of the data and accuracy of the data analysis. The corresponding author attests that all listed authors meet authorship criteria and that no others meeting the criteria have been omitted. AGS acts as guarantor for this study.

Ethical approval: Approval of the study and waiver of the requirement for individual consent were obtained from the UK Health Research Authority Confidentiality Advisory Group and London Research Ethics Committee (references 5.04(b)/10 and 10/H0720/18, respectively).

Data sharing: No additional data are available, in compliance with ethical and governance regulations under which this research was undertaken.

AGS affirms that the manuscript is an honest, accurate, and transparent account of the study being reported; that no important aspects of the study have been omitted; and that any discrepancies from the study as planned (and, if relevant, registered) have been explained.

## References

[1] Fathalla MF Incessant ovulation—a factor in ovarian neoplasia? Lancet 1971;2:163. 10.1016/S0140-6736(71)92335-X 4104488

[2] Henderson BE Ross R Bernstein L Estrogens as a cause of human cancer: the Richard and Hinda Rosenthal Foundation award lecture. Cancer Res 1988;48:246-53. 2825969

[3] Venn A Watson L Bruinsma F Giles G Healy D Risk of cancer after use of fertility drugs with in-vitro fertilisation. Lancet 1999;354:1586-90. 10.1016/S0140-6736(99)05203-4 10560672

[4] Brinton LA Trabert B Shalev V Lunenfeld E Sella T Chodick G In vitro fertilization and risk of breast and gynecologic cancers: a retrospective cohort study within the Israeli Maccabi Healthcare Services. Fertil Steril 2013;99:1189-96. 10.1016/j.fertnstert.2012.12.029 23375197 PMC4030547

[5] Källén B Finnström O Lindam A Nilsson E Nygren KG Olausson PO Malignancies among women who gave birth after in vitro fertilization. Hum Reprod 2011;26:253-8. 10.1093/humrep/deq307 21088017

[6] Yli-Kuha AN Gissler M Klemetti R Luoto R Hemminki E Cancer morbidity in a cohort of 9175 Finnish women treated for infertility. Hum Reprod 2012;27:1149-55. 10.1093/humrep/des031 22343550

[7] Luke B Brown MB Spector LG Cancer in women after assisted reproductive technology. Fertil Steril 2015;104:1218-26. 10.1016/j.fertnstert.2015.07.1135 26271227 PMC4630138

[8] Pappo I Lerner-Geva L Halevy A The possible association between IVF and breast cancer incidence. Ann Surg Oncol 2008;15:1048-55. 10.1245/s10434-007-9800-2 18214616

[9] Sergentanis TN Diamantaras AA Perlepe C Kanavidis P Skalkidou A Petridou ET IVF and breast cancer: a systematic review and meta-analysis. Hum Reprod Update 2014;20:106-23. 10.1093/humupd/dmt034 23884897

[10] Gennari A Costa M Puntoni M Breast cancer incidence after hormonal treatments for infertility: systematic review and meta-analysis of population-based studies. Breast Cancer Res Treat 2015;150:405-13. 10.1007/s10549-015-3328-0 25744295

[11] Rizzuto I Behrens RF Smith LA Risk of ovarian cancer in women treated with ovarian stimulating drugs for infertility. Cochrane Database Syst Rev 2013;8:CD008215. 23943232 10.1002/14651858.CD008215.pub2 PMC6457641

[12] Reigstad MM Larsen IK Myklebust TA Risk of breast cancer following fertility treatment--a registry based cohort study of parous women in Norway. Int J Cancer 2015;136:1140-8. 10.1002/ijc.29069 25042052 PMC4268160

[13] van Leeuwen FE Klip H Mooij TM Risk of borderline and invasive ovarian tumours after ovarian stimulation for in vitro fertilization in a large Dutch cohort. Hum Reprod 2011;26:3456-65. 10.1093/humrep/der322 22031719 PMC3212878

[14] Stewart LM Holman CD Finn JC Preen DB Hart R In vitro fertilization is associated with an increased risk of borderline ovarian tumours. Gynecol Oncol 2013;129:372-6. 10.1016/j.ygyno.2013.01.027 23385152

[15] Stewart LM Holman CD Aboagye-Sarfo P Finn JC Preen DB Hart R In vitro fertilization, endometriosis, nulliparity and ovarian cancer risk. Gynecol Oncol 2013;128:260-4. 10.1016/j.ygyno.2012.10.023 23116937

[16] Siristatidis C Sergentanis TN Kanavidis P Controlled ovarian hyperstimulation for IVF: impact on ovarian, endometrial and cervical cancer--a systematic review and meta-analysis. Hum Reprod Update 2013;19:105-23. 10.1093/humupd/dms051 23255514

[17] Reigstad MM Storeng R Myklebust TA Cancer risk in women treated with fertility drugs according to parity status-a registry-based cohort study. Cancer Epidemiol Biomarkers Prev 2017;26:953-62. 10.1158/1055-9965.EPI-16-0809 28108444 PMC5457348

[18] Dor J Lerner-Geva L Rabinovici J Cancer incidence in a cohort of infertile women who underwent in vitro fertilization. Fertil Steril 2002;77:324-7. 10.1016/S0015-0282(01)02986-7 11821091

[19] Jensen A Sharif H Kjaer SK Use of fertility drugs and risk of uterine cancer: results from a large Danish population-based cohort study. Am J Epidemiol 2009;170:1408-14. 10.1093/aje/kwp290 19884127

[20] Rossing MA Daling JR Weiss NS Moore DE Self SG Ovarian tumors in a cohort of infertile women. N Engl J Med 1994;331:771-6. 10.1056/NEJM199409223311204 8065405

[21] Jensen A Sharif H Frederiksen K Kjaer SK Use of fertility drugs and risk of ovarian cancer: Danish Population Based Cohort Study. BMJ 2009;338:b249. 10.1136/bmj.b249 19196744 PMC2640154

[22] Zegers-Hochschild F Adamson GD Dyer S The International Glossary on Infertility and Fertility Care, 2017. Fertil Steril 2017;108:393-406. 10.1016/j.fertnstert.2017.06.005 28760517

[23] Scotland ISD. Scottish Cancer Registry: NHS National Services Scotland; 2010 [Available from: http://www.isdscotland.org/Health-Topics/Cancer/Scottish-Cancer-Registry.asp

[24] Statistics OfN. Cancer Registrations, 1971 to 2011: Office for National Statistics; 2014 [updated 19th June 2014. Available from: http://www.ons.gov.uk/ons/rel/vsob1/cancer-statistics-registrations-england-series-mb1-/no-43-2012/stb-cancer-registrations-2012.html#tab-Cancer-registrations-1971-to-2011.

[25] Parkin DMCV, Ferlay J, Galceran J, Storm HH, Whelan SL. Comparability and Quality Control in Cancer Registration. Lyon: International Agency for Research on Cancer; 1994. Contract No: technical report No 19.

[26] Breslow NE Day NE Statistical Methods in Cancer Research. International Agency for Research on Cancer, 1987, Available from http://www.iarc.fr/en/publications/pdfs-online/stat/sp82/.

[27] Stata Corp Stata Statistical Software: Release 12. Stata Corp LP, 2013.

[28] Adamson GD Tabangin M Macaluso M de Mouzon J The number of babies born globally after treatment with the assisted reproductive technologies (ART). Fertil Steril 2013;100:S42-S 10.1016/j.fertnstert.2013.07.1807.

[29] Stewart LM Holman CD Hart R Bulsara MK Preen DB Finn JC In vitro fertilization and breast cancer: is there cause for concern? Fertil Steril 2012;98:334-40. 10.1016/j.fertnstert.2012.04.019 22633651

[30] Domar AD Conboy L Denardo-Roney J Rooney KL Lifestyle behaviors in women undergoing in vitro fertilization: a prospective study. Fertil Steril 2012;97:697-701.e1. 10.1016/j.fertnstert.2011.12.012 22217965

[31] Key TJ Verkasalo PK Banks E Epidemiology of breast cancer. Lancet Oncol 2001;2:133-40. 10.1016/S1470-2045(00)00254-0 11902563

[32] Cetin I Cozzi V Antonazzo P Infertility as a cancer risk factor - a review. Placenta 2008;29(Suppl B):169-77. 10.1016/j.placenta.2008.08.007 18790330

[33] Barry JA Azizia MM Hardiman PJ Risk of endometrial, ovarian and breast cancer in women with polycystic ovary syndrome: a systematic review and meta-analysis. Hum Reprod Update 2014;20:748-58. 10.1093/humupd/dmu012 24688118 PMC4326303

[34] Wentzensen N Poole EM Trabert B Ovarian Cancer Risk Factors by Histologic Subtype: An Analysis From the Ovarian Cancer Cohort Consortium. J Clin Oncol 2016;34:2888-98. 10.1200/JCO.2016.66.8178 27325851 PMC5012665

[35] Ovarian Cancer Research Fund Alliance (OCRFA). Ovarian Cancer Statistics**** https://ocrfa.org/patients/about-ovarian-cancer/statistics/2016/.

[36] Menon U Gentry-Maharaj A Hallett R Sensitivity and specificity of multimodal and ultrasound screening for ovarian cancer, and stage distribution of detected cancers: results of the prevalence screen of the UK Collaborative Trial of Ovarian Cancer Screening (UKCTOCS). Lancet Oncol 2009;10:327-40. 10.1016/S1470-2045(09)70026-9 19282241

[37] Schwarze JE Valdebenito P Ortega C Villa S Crosby J Pommer R Do women offered assisted reproduction technologies have a higher incidence of gynecologic cancer? A systematic review and meta-analysis. JBRA Assist Reprod 2017;21:115-9. 10.5935/1518-0557.20170026 28609278 PMC5473704

[38] Jones ME Swerdlow AJ Bias in the standardized mortality ratio when using general population rates to estimate expected number of deaths. Am J Epidemiol 1998;148:1012-7. 10.1093/oxfordjournals.aje.a009567 9829874

