# Supplementary material for: Risks of ovarian, breast, and corpus uteri cancer in women treated with assisted reproductive technology in Great Britain, 1991-2010: data linkage study including 2.2 million person years of observation
Source: BMJ. 2018 Jul 11;362:k2644. doi: 10.1136/bmj.k2644 (PMC6039832; doi:10.1136/bmj.k2644)
Supplement: Supplementary file 1 — Supplementary appendix: figures S1 and S2, boxes S1 and S2, and tables S1-S6 [file wilc043336.ww.pdf]

Supplementary appendix

Figure S1- Flow diagram of cohort records

302, 487 Human Fertilisation & Embryology (HFEA) records relating to women treated with ART, 1991-2010, were available for linkage. These were transferred to NHS-digital & National Records of Scotland for linkage to NHS central registries of England & Wales and Scotland (EWS).

13,017 duplicates

- 11,290 - 2 or more distinct unique member numbers (UMN) relating to the same individual
- 1,727 - duplicate UMNs

22,683 'No trace' on linkage to NHSCR

- 8, 854 - HFEA recorded address outside England, Wales and Scotland (EWS) (no possibility of linkage to NHSCR)
- 13,829 - EWS based women, no trace on NHSCR

3 records - noted to have possible linkage error after data quality checks

266,784 of 280,616 eligible records successfully linked.

- ***Linkage rate = 95.07% ****

* Denominator excludes duplicate records and records of women based overseas who had treatment in the UK.

242 records - contained no clinical fertility data

122 records - embarked from EWS before treatment date, thus no outcome data available

266,420 of 280,616 records successfully linked and containing both fertility and outcome data.

- 94.94%

1,678 records **-** treatment date missing

52 records – date of birth missing

8,904 - Women had a cancer diagnosis prior to the first calendar year of treatment.

255,786 of 280,616 potentially eligible records entered into the cohort analysis

- 91.15%

Box S1- Details of linkage & analysis

Details of linkage at National Health Service-Digital and National Records for Scotland

Matching was initially deterministic, matching on:-

- Forename, Surname
- 2 of 3 parts of date of birth match

If more than one match was found no match was accepted and the records would go for manual matching along with unmatched records. Manual matching utilised: -

- Forename, Surname, Date of birth
- Other recorded names
- Place of birth
- Treatment centre/ Cycle date

Quality control process: -

Automatic matching algorithm used (A) was tested against an algorithm using exact date of birth match (B). Both A and B were performed on 4239 cases. The 4239 cases then underwent manual matching gold standard.

A – 0/4239 false positive matches

B – 9/4239 false positive matches

Additional details of analysis: -

As detailed in the main paper, expected cancers were calculated by multiplying person-years at risk by corresponding national incidence rates (by 5-year age band and individual calendar year) for the general population of England & Wales. Annual national incidence rates 1991-1998 are for England and Wales, thereafter national rates refer to England only as rates were not published for England and Wales.

Table S1. Meta-data for available HFEA variables (linked cohort)

HFEA data item	Frequency	Details	Data Source	% complete
**Date of Birth**	255,786		Self-reported to HFEA	100
**Ethnic group**	46,107	30 potential responses	Self-reported to HFEA	18.0
**Start date of first treatment cycle**	255,786	Year of each treatment cycle recorded. Year mid-point used to calculate person-years at risk.	Clinic reported to HFEA	100
**Start date of last treatment cycle**	255,786		Clinic reported to HFEA	100
**Age at first treatment**	255,786	Categorised: -<br><25 yrs- 5,671<br>25-29 yrs- 39,932<br>30-34 yrs- 92,788<br>35-39 yrs- 85,868<br>40-44 yrs- 28,174<br>45+yrs – 3,353	Derived, first treatment cycle date minus date of birth	100
**Broad Cause of infertility**	244,286	Female- 70,293<br>Male- 84,871<br>Both- 41,365<br>Unexplained- 47,757<br>Unrecorded- 11,500 & all specific causes negative.	Clinic reported to HFEA	95.5
**Endometriosis**	255,786	Yes- 18,630<br>No- 237,156	Clinic reported to HFEA	100
**Tubal disease**	255,786	Yes- 66,370<br>No- 189,416	Clinic reported to HFEA	100
**Ovulatory disorder**	255,786	Yes- 36,016<br>No- 219,770	Clinic reported to HFEA	100
**Male factor infertility**	255,786	Any Yes-126,236 No-129,550<br>Sperm concentration<br>Yes- 18,679 No-237,107<br>Sperm morphology<br>Yes-10,586 No-245,200<br>Sperm motility<br>Yes-9,263 No-246,523<br>Sperm immune issue<br>Yes-2,493 No-253,293	Clinic reported to HFEA	
**Primary Female infertility**	255,576	Yes-113,918<br>No- 141,658<br>Unrecorded-210	Clinic reported to HFEA	99.9
**Secondary Female Infertility**	255,786	Yes-86,322<br>No-169,464	Clinic reported to HFEA	100
**Primary Male Infertility**	255,786	Yes-117,207<br>No-138,579	Clinic reported to HFEA	100
**Secondary Male Infertility**	255,786	Yes-80,843<br>No-174,943	Clinic reported to HFEA	100
**Primary Couple infertility**	255,786	Yes-139,272<br>No-116,514	Clinic reported to HFEA	100
**Secondary Couple infertility**	255,786	Yes-58,584<br>No-197,202	Clinic reported to HFEA	100
**Duration of infertility**	206,304	<2yrs- 17,194<br>2-3yrs- 67,529<br>4-5yrs- 56,203<br>6-7yrs- 29,946<br>8-9yrs - 15,394<br>>=10yrs -20,038<br>Unrecorded- 49,482	Self-reported to HFEA	80.6

**Number of Treatment cycles**	255,778	Natural cycle only-9,781<br>Stimulated cycles-<br>1- 131,670<br>2- 63,842<br>3-4- 41,224<br>5+ - 9,261<br>Unrecorded - 8	Clinic reported to HFEA	99.9
**Type of ART treatment**	255,177	IVF only- 150,700<br>ICSI/ Unspecified micromanipulation- 76, 596<br>IVF & ICSI- 27,881	Clinic reported to HFEA	99.8
**Treatment centre**	255,786	Treatment centre only geographical variable available	Clinic reported to HFEA	100
**Number of Pregnancies by end of last treatment cycle**	255,377	0- 82,747<br>1- 94,836<br>2-3- 63,821<br>4-5- 11,246<br>6+ 2,727<br>Unknown - 409	Derived variable from self - reported pregnancies on registration of last treatment cycle plus HFEA recorded ART pregnancies from last treatment cycle (validated against HFEA recorded ART pregnancies from previous cycles).	99.8
**Years since last pregnancy**	121,698	Variable contains a number of values which are likely to be age at last pregnancy.	Self-reported to HFEA	47.6
**Age at last pregnancy**	121,698	Median- 31.7 yrs<br>IQR 35.5-27.7 yrs	Self-reported to HFEA	47.6
**Number of live births by end of last treatment cycle**	255,701	0- 129,217<br>1- 96,839<br>2-3- 27,593<br>4+ 2,052<br>Unrecorded- 85	Derived variable from self - reported births on registration of last treatment cycle plus HFEA recorded ART birth from last treatment cycle (validated against HFEA recorded ART births from previous cycles).	99.9
**Multiple births**	255,786	Yes- 29,366<br>No- 29,366	Clinic reported to HFEA	100
**ART birth recorded by HFEA**	255,786	Yes- 105,183<br>No-150,183	Clinic reported to HFEA	100

Figure S2- Person years of follow up within the cohort of women who had assisted conception, by year of first treatment

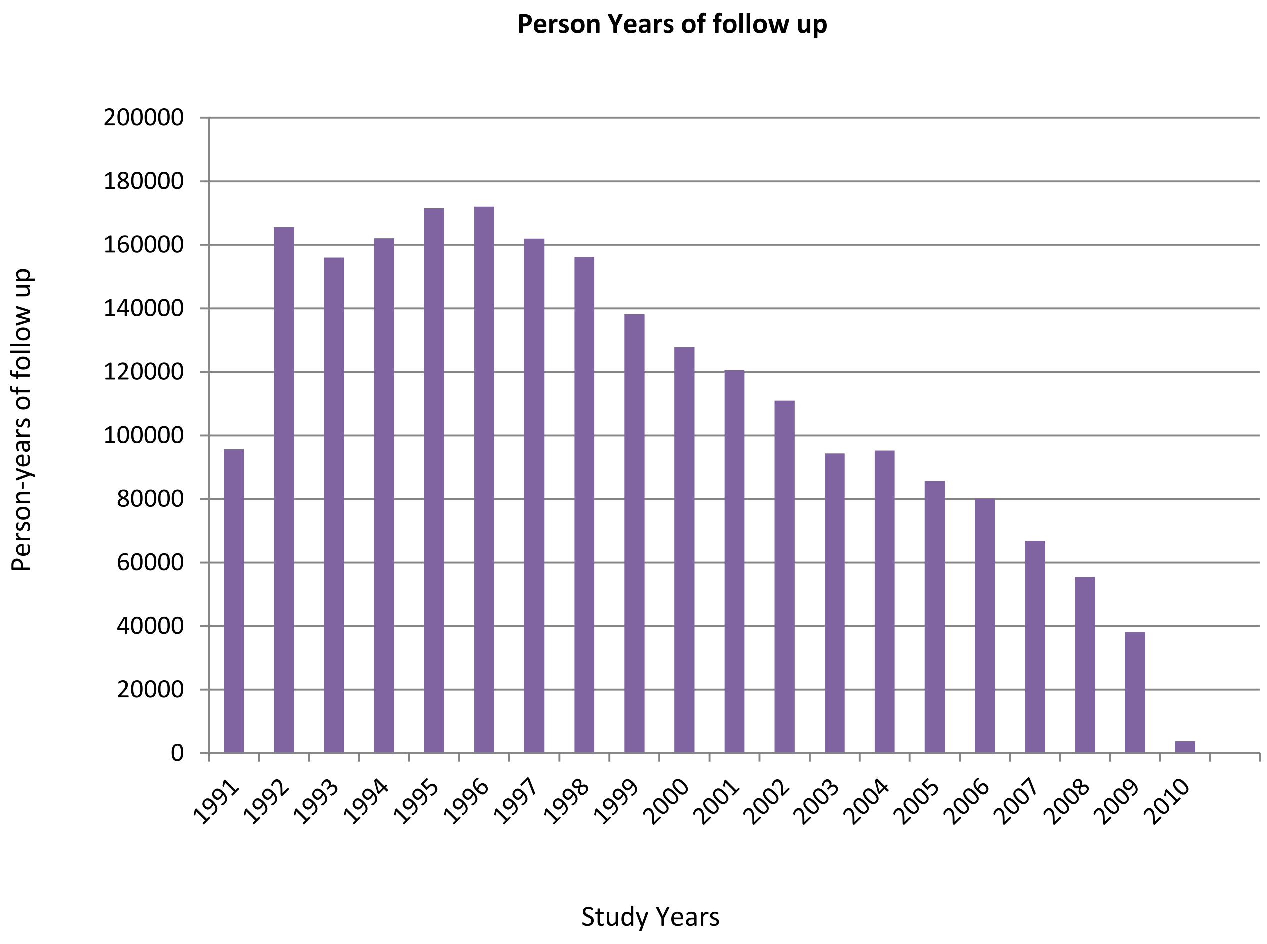

Table S2- Cohort frequency and person years of follow up by year of first

Year of first treatment	No. of women	%	Cumulative %	Person-years of follow-up	Person-years as % of total	Cumulative person years as % of cohort
1991	5,047	2.0	1.97	95654	4.2	4.2
1992	9,169	3.6	5.56	165557	7.3	11.6
1993	9,095	3.6	9.11	155957	6.9	18.5
1994	9,996	3.9	13.02	162009	7.2	25.7
1995	11,216	4.4	17.41	171479	7.6	33.3
1996	12,004	4.7	22.10	172036	7.6	40.9
1997	12,124	4.7	26.84	161905	7.2	48.1
1998	12,578	4.9	31.76	156201	6.9	55.0
1999	12,058	4.7	36.47	138110	6.1	61.1
2000	12,149	4.8	41.22	127788	5.7	66.8
2001	12,622	4.9	46.15	120568	5.3	72.1
2002	12,932	5.1	51.21	110950	4.9	77.0
2003	12,414	4.9	56.06	94329	4.2	81.2
2004	14,368	5.6	61.68	95264	4.2	85.4
2005	15,144	5.9	67.60	85673	3.8	89.2
2006	17,130	6.7	74.30	80136	3.6	92.7
2007	18,070	7.1	81.36	66809	3.0	95.7
2008	20,494	8.0	89.38	55464	2.5	98.2
2009	22,068	8.6	98.00	38141	1.7	99.9
2010	5,108	2.0	100.00	3760	0.2	100.0
Total	255,786	100.0	100.00	2257789	100.0	100.0

Table S3- Sensitivity analysis excluding the first 12 months of follow up for breast, corpus uteri and ovarian cancer

Factor	Person-years follow-up	Type of Cancer					
		Breast‡		Corpus Uteri§		Ovarian‖	
		Observed Cancers	SIR (95%CI)	Observed Cancers	SIR (95%CI)	Observed Cancers	SIR (95%CI)
Overall	2,004,121	2384	0.95 (0.92 to 0.99)	157	1.12 (0.94 to 1.30)	356	1.31 (1.18 to 1.45)
Age at first treatment (years)							
<25	42,574	11	1.05 (0.53 to 1.88)	0	0.00 (0.00 to 7.13)	<5	#
25 to 29	342,334	171	0.87 (0.74 to 1.01)	8	1.01 (0.44 to 2.00)	55	1.99 (1.50 to 2.59)
30-34	774,230	723	0.92 (0.86 to 0.99)*	41	1.16 (0.83 to 1.58)	124	1.42 (1.18 to 1.69)
35-39	628,952	955	0.95 (0.89 to 1.02)	70	1.23 (0.96 to 1.55)	118	1.16 (0.96 to 1.39)
40-44	190,890	436	1.01 (0.92 to 1.11)	32	0.97 (0.67 to 1.37)	47	1.07 (0.78 to 1.42)
45+	25,142	88	1.13 (0.91 to 1.39)	6	0.71 (0.26 to 1.56)	<10	#
		Trend across categories P=0.03		Trend across categories P=0.54		Trend across categories P=0.001	
Infertility cause							
Any female factor	998,634	1224	0.95 (0.90 to 1.00)	95	1.26 (1.02 to 1.54)	221	1.58 (1.38 to 1.81)
Male factor only	672,834	727	0.91 (0.85 to 0.98)	40	0.92 (0.66 to 1.25)	88	1.01 (0.81 to 1.24)
Unexplained	279,249	377	1.08 (0.98 to 1.20)	16	0.83 (0.47 to 1.35)	33	0.88 (0.60 to 1.23)
Unrecorded	53,406	56	0.87 (0.66 to 1.13)	6	1.67 (0.61 to 3.64)	14	1.98 (1.08 to 3.33)
History of endometriosis							
Yes	162,795	204	0.98 (0.85 to 1.12)	9	0.78 (0.35 to 1.47)	49	2.19 (1.62 to 2.89)
No	1,841,327	2180	0.95 (0.91 to 0.99)	148	1.14 (0.96 to 1.34)	307	1.23 (1.10 to 1.38)
History of tubal disease							
Yes	644,518	800	0.97 (0.90 to 1.04)	57	1.21 (0.92 to 1.57)	151	1.69 (1.44 to 1.99)
No	1,359,603	1584	0.95 (0.90 to 0.99)	100	1.06 (0.86 to 1.28)	205	1.12 (0.97 to 1.29)
History of ovulatory problems							
Yes	275,753	333	0.91 (0.82 to 1.02)	38	1.65 (1.17 to 2.27)	43	1.08 (0.78 to 1.45)
No	1,728,369	2051	0.96 (0.92 to 1.00)	119	1.00 (0.83 to 1.20)	313	1.35 (1.20 to 1.51)
Duration of infertility at last treatment cycle (years)							
< 2	116,371	142	0.84 (0.71 to 1.00)	<5	## *	23	1.26 (0.82 to 1.93)
2-3	373,788	481	1.04 (0.95 to 1.14)	22	0.83 (0.52 to 1.26)	62	1.23 (0.94 to 1.57)
4-5	392,584	498	1.00 (0.92 to 1.10)	30	1.07 (0.73 to 1.54)	66	1.23 (0.95 to 1.57)
6-7	242,061	298	0.91 (0.81 to 1.02)	27	1.43 (0.94 to 2.07)	57	1.63 (1.24 to 2.12)
8-9	136,379	185	0.94 (0.81 to 1.08)	16	1.38 (0.79 to 2.24)	33	1.60 (1.10 to 2.24)

10+	189,948	305	0.94 (0.84 to 1.05)	37	1.72 (1.21 to 2.38)	50	1.49 (1.10 to 1.96)
Unrecorded	275,893	360	1.06 (0.95 to 1.17)	<20	#	36	0.99 (0.69 to 1.37)
		Trend across categories P=0.47		Trend across categories P<0.001		Trend across categories P=0.13	
Total number of stimulated cycles							
0 – 'natural cycle' only	81,304	136	0.90 (0.76 to 1.07)	8	0.68 (0.30 to 1.35)	15	0.94 (0.53 to 1.55)
1	912,394	1107	0.96 (0.90 to 1.01)	85	1.29 (1.03 to 1.59)	174	1.39 (1.19 to 1.61)
2	410,483	545	1.01 (0.93 to 1.10)	29	0.95 (0.64 to 1.37)	77	1.33 (1.05 to 1.67)
3-4	265,687	381	1.01 (0.91 to 1.11)	24	1.12 (0.72 to 1.66)	48	1.22 (0.90 to 1.61)
5+	57,107	100	1.10 (0.90 to 1.34)	5	0.94 (0.31 to 2.19)	13	1.41 (0.75 to 2.41)
Unrecorded	50	0	0.00 (0.0 to 33.29)	0	0.00 (0.0 to 299.57)	0	0.00 (0.0 to 299.57)
		Trend across categories P=0.13		Trend across categories P=0.81		Trend across categories P=0.95	
Total number of live births at last cycle completion							
0	882,844	1166	0.95 (0.89 to 1.00)	116	1.60 (1.32 to 1.92)	189	1.45 (1.25 to 1.67)
1	623,485	801	1.04 (0.97 to 1.12)	24	0.56 (0.36 to 0.83)	109	1.31 (1.07 to 1.58)
2+	220,364	301	0.94 (0.84 to 1.05)	11	0.56 (0.28 to 1.00)	29	0.86 (0.57 to 1.23)
Unrecorded	332	1	2.13 (0.05 to 11.86)	0	0.00 (0.0 to 99.86)	0	0.00 (0.0 to 59.92)
		Trend across categories P=0.48		Trend across categories P<0.001		Trend across categories P=0.01	
Multiple birth as recorded at last cycle completion							
Yes	203,766	253	1.15 (1.01 to 1.30)	5	0.44 (0.14 to 1.03)	31	1.26 (0.86 to 1.79)
No	1,523,258	2016	0.96 (0.92 to 1.00)	146	1.18 (1.00 to 1.39)	296	1.33 (1.18 to 1.49)
Time since last treatment (years)							
0-3	435,973	337	0.99 (0.88 to 1.10)	22	1.58 (0.99 to 2.39)	56	1.32 (1.00 to 1.71)
3-6	486,191	529	1.04 (0.95 to 1.13)	29	1.28 (0.85 to 1.83)	73	1.27 (1.00 to 1.60)
6-10	444,324	657	1.00 (0.93 to 1.08)	38	1.07 (0.76 to 1.47)	84	1.24 (0.99 to 1.53)
10-15	296,445	590	0.93 (0.86 to 1.01)	45	0.99 (0.72 to 1.33)	86	1.39 (1.11 to 1.71)
15+	64,091	156	0.86 (0.73 to 1.01)	17	0.98 (0.57 to 1.57)	28	1.57 (1.04 to 2.27)
		Trend across categories P=0.06		Trend across categories P=0.06		Trend across categories P=0.46	

Table S4- Sensitivity analysis excluding the first 12 months of follow up for invasive and borderline ovarian tumours

Factor	Person-years follow-up	Invasive Ovarian Tumours§§		Borderline Ovarian Tumors‖‖	
		Observed Cancers	SIR (95%CI)	Observed Cancers	SIR (95%CI)
Overall	2,004,121	244	1.37 (1.21 to 1.56)	112	1.19 (0.98 to 1.43)
Age at first treatment (years)					
<25	42,574	<5	#	<5	#
25-29	342,334	32	2.24 (1.53 to 3.16)	23	1.72 (1.09 to 2.58)
30-34	774,230	76	1.44 (1.13 to 1.80)	48	1.39 (1.02 to 1.84)
35-39	628,952	87	1.25 (1.01 to 1.55)	31	0.96 (0.65 to 1.37)
40-44	190,890	39	1.17 (0.83 to 1.60)	8	0.74 (0.32 to 1.46)
45+	25,142	<10	#	<5	#
		Trend across categories P=0.01		Trend across categories P=0.01	
Infertility cause					
Any female factor	998,634	155	1.67 (1.42 to 1.96)	66	1.40 (1.09 to 1.79)
Male factor only	672,834	60	1.06 (0.81 to 1.37)	28	0.90 (0.60 to 1.30)
Unexplained	279,249	20	0.82 (0.50 to 1.27)	13	0.98 (0.52 to 1.67)
Unrecorded	53,406	9	1.99 (0.91 to 3.78)	5	1.98 (0.64 to 4.61)
History of endometriosis					
Yes	162,795	37	2.51 (1.77 to 3.47)	12	1.57 (0.81 to 2.73)
No	1,841,327	207	1.27 (1.10 to 1.45)	100	1.16 (0.94 to 1.41)
History of tubal disease					
Yes	644,518	101	1.72 (1.40 to 2.09)	50	1.65 (1.23 to 2.18)
No	1,359,603	143	1.20 (1.01 to 1.42)	62	0.97 (0.75 to 1.25)
History of ovulatory problems					
Yes	275,753	32	1.19 (0.82 to 1.61)	11	0.84 (0.42 to 1.51)
No	1,728,369	212	1.40 (1.22 to 1.61)	101	1.25 (1.02 to 1.52)
Duration of infertility at last treatment cycle (years)					
< 2	116,371	15	1.23 (0.69 to 2.02)	8	1.41 (0.61 to 2.79)
2-3	373,788	47	1.43 (1.05 to 1.90)	15	0.85 (0.48 to 1.41)
4-5	392,584	49	1.40 (1.03 to 1.85)	17	0.92 (0.54 to 1.47)
6-7	242,061	39	1.67 (1.19 to 2.29)	18	1.55 (0.92 to 2.45)
8-9	136,379	25	1.78 (1.15 to 2.63)	8	1.21 (0.52 to 2.37)

10+	189,948	35	1.46 (1.02 to 2.03)	15	1.55 (0.87 to 2.55)
Unrecorded	275,893	21	0.90 (0.56 to 1.38)	15	1.13 (0.63 to 1.87)
		Trend across categories P=0.39		Trend across categories P=0.17	
Total number of stimulated cycles					
0 – ‘natural cycle’ only	81,304	13	1.11 (0.59 to 1.90)	<5	#
1	912,394	118	1.43 (1.19 to 1.72)	56	1.30 (0.98 to 1.69)
2	410,483	50	1.32 (0.98 to 1.73)	27	1.37 (0.90 to 1.99)
3-4	265,687	39	1.48 (1.05 to 2.02)	9	0.69 (0.32 to 1.31)
5+	57,107	11	1.74 (0.87 to 3.11)	<5	#
		Trend across categories P=0.48		Trend across categories P=0.30	
Total number of live births at last cycle completion					
0	882,844	136	1.55 (1.30 to 1.84)	53	1.24 (0.93 to 1.62)
1	623,485	77	1.42 (1.12 to 1.78)	32	1.09 (0.75 to 1.54)
2+	220,364	18	0.78 (0.46 to 1.23)	11	1.01 (0.51 to 1.81)
Unrecorded	332	0	0.00 (0.00 to 99.86)	0	0.00 (0.00 to 149.8)
		Trend across categories P=0.01		Trend across categories P=0.46	
Multiple birth as recorded at last cycle completion					
Yes	203,766	21	1.37 (0.85 to 2.09)	10	1.08 (0.52 to 1.99)
No	1,523,258	210	1.41 (1.22 to 1.61)	86	1.17 (0.93 to 1.44)
Time since last treatment (years)					
0-3	435,973	39	1.62 (1.15 to 2.21)	17	0.93 (0.54 to 1.48)
3-6	486,191	45	1.27 (0.93 to 1.71)	28	1.27 (0.85 to 1.84)
6-10	444,324	63	1.37 (1.05 to 1.75)	21	0.96 (0.59 to 1.46)
10-15	296,445	63	1.38 (1.06 to 1.77)	23	1.39 (0.88 to 2.08)
15+	64,091	21	1.52 (0.94 to 2.32)	7	1.75 (0.70 to 3.60)
		Trend across categories P=0.85		Trend across categories P=0.21	

Table S5- Sensitivity analysis excluding the first 12 months of follow up for invasive and in-situ breast cancer

Factor	Person-years follow-up	Invasive breast cancer[††]		In situ- breast cancer[‡‡]	
		Observed Cancers	SIR (95%CI)	Observed Cancers	SIR (95%CI)
Overall	2,004,121	2089	0.93 (0.89 to 0.97)	280	1.15 (1.02 to 1.29)
Age at first treatment (years)					
<25	42,574	11	1.14 (0.57 to 2.04)	0	0.00 (0.00 to 4.41)
25-29	342,334	154	0.85 (0.72 to 1.00)	16	1.12 (0.64 to 1.81)
30-34	774,230	635	0.89 (0.82 to 0.96)	84	1.29 (1.03 to 1.59)
35-39	628,952	850	0.95 (0.89 to 1.02)	97	0.95 (0.77 to 1.16)
40-44	190,890	373	0.99 (0.89 to 1.09)	61	1.19 (0.91 to 1.53)
45+	25,142	66	0.99 (0.76 to 1.26)	22	2.06 (1.29 to 3.12)
		Trend across categories P=0.07		Trend across categories P=0.67	
Infertility cause					
Any female factor	998,634	1068	0.93 (0.87 to 0.98)	146	1.14 (0.96 to 1.34)
Male factor only	672834	632	0.88 (0.81 to 0.95)	90	1.18 (0.95 to 1.45)
Unexplained	279,249	337	1.08 (0.97 to 1.20)	<45	#
Unrecorded	53,406	52	0.90 (0.67 to 1.18)	<5	#
History of endometriosis					
Yes	162,795	176	0.94 (0.81 to 1.09)	26	1.28 (0.84 to 1.88)
No	1,841,327	1913	0.93 (0.89 to 0.97)	254	1.13 (1.00 to 1.28)
History of tubal disease					
Yes	644,518	701	0.95 (0.88 to 1.02)	90	1.11 (0.89 to 1.36)
No	1,359,603	1388	0.92 (0.87 to 0.97)	190	1.17 (1.01 to 1.34)
History of ovulatory problems					
Yes	275,753	294	0.90 (0.80 to 1.01)	38	1.01 (0.72 to 1.39)
No	1,728,369	1795	0.94 (0.89 to 0.98)	242	1.17 (1.03 to 1.33)
Duration of infertility at last treatment cycle (years)					
< 2	116,371	128	0.85 (0.71 to 1.02)	14	0.80 (0.44 to 1.34)
2-3	373,788	422	1.02 (0.92 to 1.12)	57	1.25 (0.95 to 1.63)
4-5	392,584	439	0.99 (0.90 to 1.08)	52	1.08 (0.80 to 1.41)
6-7	242,061	260	0.88 (0.78 to 1.00)	35	1.07 (0.75 to 1.49)
8-9	136,379	159	0.90 (0.77 to 1.06)	25	1.25 (0.81 to 1.85)

10+	189,948	262	0.91 (0.80 to 1.03)	42	1.19 (0.86 to 1.60)
Unrecorded	275,893	311	1.02 (0.91 to 1.14)	48	1.49 (1.10 to 1.97)
		Trend across categories P=0.30		Trend across categories P=0.53	
Total number of stimulated cycles					
0 – ‘natural cycle’ only	81,304	115	0.87 (0.72 to 1.04)	21	1.20 (0.74 to 1.83)
1	912,394	981	0.95 (0.89 to 1.01)	117	1.03 (0.85 to 1.23)
2	410,483	472	0.97 (0.89 to 1.07)	70	1.32 (1.03 to 1.66)
3-4	265,687	334	0.99 (0.88 to 1.10)	45	1.19 (0.87 to 1.60)
5+	57,107	79	0.98 (0.77 to 1.22)	20	2.14 (1.31 to 3.31)
Unrecorded		0	0.00 (0.0 to 37.45)	0	0.00 (0.0 to 299.57)
		Trend across categories P=0.27		Trend across categories P=0.03*	
Total number of live births at last cycle completion	882,844				
0	623,485	1027	0.94 (0.88 to 0.99)	129	1.04 (0.87 to 1.23)
1	220,364	691	1.00 (0.93 to 1.08)	106	1.43 (1.17 to 1.72)
2+	332	263	0.92 (0.81 to 1.04)	37	1.12 (0.79 to 1.55)
Unrecorded		0	0.00 (0.0 to 7.13)	1	20.00 (0.51 to 111.43)
		Trend across categories P=0.71		Trend across categories P=0.21	
Any multiple birth as recorded at last cycle completion	203,766				
Yes	1,523,258	230	1.16 (1.01 to 1.32)	21	1.04 (0.65 to 1.59)
No		1751	0.93 (0.89 to 0.98)	252	1.19 (1.05 to 1.35)
Time since last treatment (years)	435,973				
0-3	486,191	307	0.98 (0.87 to 1.09)	30	1.18 (0.80 to 1.69)
3-6	444,324	476	1.03 (0.94 to 1.12)	51	1.24 (0.93 to 1.63)
6-10	296,445	556	0.94 (0.87 to 1.02)	95	1.52 (1.23 to 1.85)
10-15	64,091	510	0.93 (0.85 to 1.01)	75	0.98 (0.77 to 1.22)
15+		132	0.86 (0.72 to 1.02)	22	0.85 (0.54 to 1.29)
		Trend across categories P=0.07		Trend across categories P=0.07	

Table S6- Risk of any ovarian cancer, invasive and borderline ovarian tumours in women with and without endometriosis and or nulliparity, stratified by age at first treatment

Factor	Person-years follow-up	Type of ovarian tumour					
		All ovarian tumours[‖]		Invasive ovarian tumour[§§]		Borderline ovarian tumour[‖‖]	
		Observed Cancers	SIR (95%CI)	Observed Cancers	SIR (95%CI)	Observed Cancers	SIR (95%CI)
Age at first treatment if at least one risk factor (endometriosis, nulliparity) recorded							
<25 years	25,787	<5	#	<5	#	<5	#
25-29 years	197,309	44	2.84 (2.06 to 3.81)	26	3.28 (2.14 to 4.80)	18	2.37 (1.40 to 3.74)
30-34 years	448,040	97	1.97 (1.60 to 2.40)	55	1.86 (1.40 to 2.43)	42	2.13 (1.54 to 2.88)
35-39 years	399,110	93	1.50 (1.21 to 1.84)	70	1.67 (1.30 to 2.11)	23	1.15 (0.73 to 1.72)
40-44 years	137,314	33	1.11 (0.76 to 1.56)	24	1.09 (0.70 to 1.61)	9	1.17 (0.54 to 2.23)
45+ years	13,233	<5	#	<5	#	<5	#
		Trend across categories P<0.001		Trend across categories P<0.001		Trend across categories P=0.01	
Age at first treatment if no risk factors recorded							
<25 years	22,400	<5	#	<5	#	<5	#
25-29 years	184,655	20	1.42 (0.87 to 2.19)	<10	#	11	1.57 (0.78 to 2.80)
30-34 years	418,312	45	1.02 (0.75 to 1.37)	26	1.01 (0.66 to 1.48)	19	1.05 (0.63 to 1.63)
35-39 years	314,946	41	0.87 (0.62 to 1.18)	27	0.86 (0.57 to 1.25)	14	0.89 (0.49 to 1.50)
40-44 years	81,453	17	0.94 (0.55 to 1.51)	16	1.19 (0.68 to 1.93)	<5	#
45+ years	15,231	<10	#	5	1.21 (0.39 to 2.82)	<5	#
		Trend across categories P=0.07		Trend across categories P=0.62		Trend across categories P=0.02	

Box S2- Investigation of women with unrecorded cause of infertility

Investigation of women with unrecorded cause of infertility (n= 11,500)

Women with unrecorded cause of infertility had significantly increased rates of breast, ovarian and corpus uteri cancer. Reasons for this are unclear. Those with unrecorded cause of infertility had treatment more recently, at older ages, with fewer cycles, shorter duration of infertility, more 'freeze-all' cycles (data for 'freeze-all' cycles are available for only a sub-set of our cohort; women who had children after assisted conception between 1992 and 2008). Women with unrecorded cause of infertility had a higher cancer incidence within the first 12 months.

Variable	Whole cohort average (95%CI)	Unrecorded cause of infertility cohort average (95%CI)	Test statistic
First treatment year	2002.0 (2002.0 to 2002.1)	2005.5 (2005.4 to 2005.5)	P<0.001
Age at first treatment (years)	34.4 (34.4 to 34.4)	36.3 (36.2 to 36.4)	P<0.001
Number of treatment cycles	1.77 (1.76 to 1.77)	1.51 (1.49 to 1.53)	P<0.001
Duration of infertility at last treatment cycle	4.90 (4.89 to 4.92)	3.69 (3.62 to 3.77)	P<0.001
'Freeze -all' cycle	11.9% (11.7 to 12.1)	13.2% (12.1 to 14.8)	-
Proportion of cancers diagnosed within 12months of first treatment.	6.2% (5.3 to 7.0)	45.7% (37.5 to 54.0)	P<0.001

Therefore excess risk in this sub-group might be due to reverse causation; cancer and/or related treatment causing infertility rather than arising as a result of infertility or its treatment. Whilst we excluded all women with a cancer diagnosis in calendar years before first treatment year, we could not exclude women diagnosed in the same calendar year as first treatment because exact treatment date was unavailable. As some results remained significant after excluding the first 12 months of follow up, there may be further explanations. Unfortunately study regulations preclude inspection of clinical notes to investigate further.

Table Legends for appendix

Cohort restricted to women who underwent assisted reproduction who were cancer free at least for the first 12 months after the first cycle.

† Absolute Excess Risk per 100,000 person years at risk

‡ 'Breast Cancer'= ICD-9: 1740-9, 2330, 2383; ICD-10: C500-9, D050-9, D486

§ 'Corpus Uteri Cancer'= ICD-9: 1820-8; ICD-10: C54

‖ 'Ovarian Cancer'= ICD-9: 1830-1839, 2362; ICD-10: C56, C570-C574, C481, C482, D391

¶ See Supplemental Data for results excluding the first 12 months of follow up.

†† 'Invasive Breast Cancer'= ICD-9: 1740-9: ICD-10:C500-9

‡‡ 'In-situ Breast Cancer'= ICD-9: 2330: ICD-10: D050-9

§§ 'Invasive Ovarian Tumours'= ICD-9: 1830-1839 (excluding morphology codes 8442/8451/8462/8472/8473) 2362; ICD-10: C56, C570-C574, C481, C482 (excluding morphology codes 8442/8451/8462/8472/8473).

‖‖ 'Borderline Ovarian Tumours'=ICD-9 1830 (with morphology codes 8442/8451/8462/8472/8473); ICD-10 D391, C56 (with morphology codes 8442/8451/8462/8472/8473).

SIRs suppressed to comply with data disclosure regulations where cells relate to small numbers of individuals. None of the SIRs for affected cells approached significance.

SIR suppressed to comply with data disclosure regulations where cells relate to small numbers of individuals. SIR was significantly lower than age standardised expectation (P=0.014).
